# The pancreatic β-cell incretin response is modulated by mitochondrial transaminase GPT2

**DOI:** 10.21203/rs.3.rs-6950998/v1

**Published:** 2025-06-30

**Authors:** Sabyasachi Sen, Andrea V. Rozo, Matthew W. Haemmerle, Jeffrey Roman, Andrea V. Scota, Xiaodun Yang, Christine A. Juliana, Sarah A. Tersey, Eric M. Morrow, Nicolai M. Doliba, Doris A. Stoffers

**Affiliations:** 1 Institute for Diabetes, Obesity, and Metabolism, Perelman School of Medicine, University of Pennsylvania, Philadelphia, PA, USA,; 2 Division of Endocrinology and Diabetes, The Children’s Hospital of Philadelphia, Philadelphia, PA, USA,; 3 Section of Endocrinology, Diabetes and Metabolism, Department of Medicine, The University of Chicago, Chicago, IL, USA,; 4 Department of Molecular Biology, Cell Biology and Biochemistry, Brown University, Providence, RI, USA

## Abstract

The effect of the incretin hormones GLP-1 and GIP to promote pancreatic β-cell function is exploited by an expansive menu of incretin mimetics for the treatment of type 2 diabetes (T2D); however, the incretin effect is well known to diminish as T2D progresses. Here, we show that silencing of stress-inducible mitochondrial protein glutamic pyruvate transaminase 2 (GPT2) enhances the β-cell incretin response. Mice with β-cell specific *Gpt2* deficiency (*Gpt2*^βKO^) have improved oral glucose tolerance and insulin secretion due to enhanced β-cell incretin sensitivity. In the diet induced obesity (DIO) model of T2D, *Gpt2*^βKO^ mice maintained lower non-fasting glucose and improved oral glucose tolerance and insulin secretion. The effect of GLP-1 receptor (GLP-1R) agonism on β-cell survival was also enhanced in *Gpt2*^βKO^ islets. GPT2 was markedly induced in human islets from donors with type 2 diabetes and in non-diabetic donor islets exposed to glucolipotoxicity. Silencing *GPT2* in human β-cells enhanced β-cell incretin sensitivity and survival, and it reversed incretin unresponsiveness in T2D islets. These findings raise GPT2 as a therapeutic target to mitigate β-cell dysfunction in T2D.

## Introduction

The actions of two gut derived polypeptides: GIP (glucose-dependent insulinotropic polypeptide) and GLP-1 (glucagon-like peptide-1), commonly referred to as ‘incretin’ hormones^1^, are responsible for more than 50% of postprandial insulin output from pancreatic β-cells^2–5^. These peptides act on β-cells through their cognate G protein–coupled receptor GPCRs to potentiate glucose-stimulated insulin secretion while simultaneously promoting β-cell survival and replication^6–8^. Progressive loss of functional β-cell mass^9–11^ and decline in incretin sensitivity^12–14^ are central to pancreatic β-cell failure in type 2 diabetes (T2D). Hyperglycemia and high levels of circulating free fatty-acid have been shown to promote β-cell apoptosis and contribute to loss of incretin sensitivity^15–17^. GLP-1R agonists exploit this physiology to counter the effects of glucolipotoxicity (GLT) and improve beta cell function and survival^18–20^. However, the molecular mechanisms underlying these effects remains poorly understood^21^.

We previously identified *GPT2* as a stress-induced gene in both human and mouse islets whose silencing in mouse β-cells *ex vivo* protects from ER stress induced apoptosis^22^. *GPT2* encodes a mitochondrial aminotransferase that catalyzes the bidirectional conversion of pyruvate and glutamate to alanine and a-ketoglutarate. It has been implicated in glutamine mediated TCA-cycle anaplerosis in several cancers^23–25^ and primary neurons^26–28^.

Here, we find that GPT2 modulates the sensitivity of both mouse and human β cells to incretin hormones. Mice with β-cell-specific *Gpt2* deficiency exhibit enhanced incretin stimulated insulin secretion and cell survival when maintained on regular chow and during high fat diet feeding. GPT2 expression is markedly upregulated in islets from donors with T2D and induced in non-diabetic human islets exposed to GLT. shRNA mediated silencing of *GPT2* in the β-cells of human islets from non-diabetic donors enhances sensitivity to the GLP-1R agonist Exendin-4 (Ex4) and alleviates GLT induced apoptosis. Of high translational relevance, silencing of *GPT2* in the β-cells of human T2D islets restored glucose and Ex4 responsiveness, raising GPT2 as a therapeutic target to mitigate β-cell dysfunction in diabetes.

## Results

### Heightened response of Gpt2^βKO^ mice to the GLP-1 receptor agonist Exendin-4

**Figure 1.**
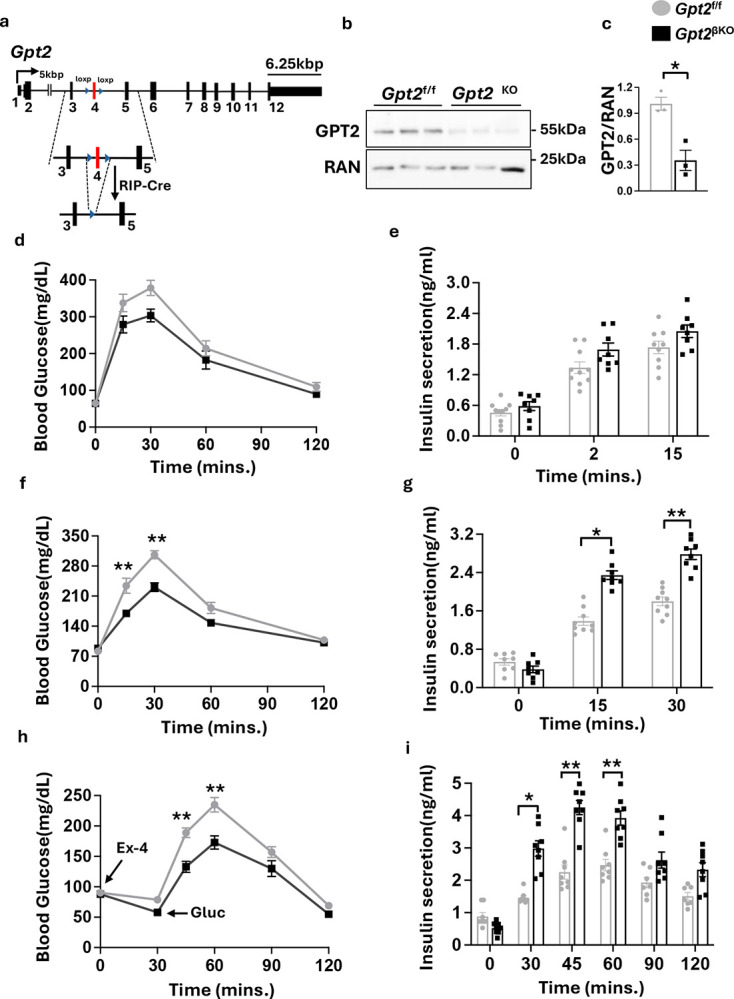
Enhanced incretin effect in *Gpt2*^βKO^ mice. **a**. Schematic showing floxed allele before and after Cre mediated recombination between loxP sites leads to the removal of exon 4 of the *Gpt2* gene. **b**. Western blot analysis showing GPT2 levels in mouse islet preparations from 3 *Gpt2*^f/f^ and *Gpt2*^βKO^ mice. **c**. Densitometric quantification of signal intensity normalized to RAN. **d**. Intraperitoneal glucose tolerance test assessed in 8–10-week-old, chow-fed male *Gpt2*^f/f^ or *Gpt2*^βKO^ mice. **e**. Intraperitoneal glucose stimulated insulin secretion (GSIS) at 10–12 weeks of age. **f**. Oral glucose tolerance test at 9–11-weeks of age. **g**. Oral GSIS at 11–13 weeks. 2g/kg BW glucose was administered for GTTs and 3g/kg BW glucose for GSIS assays. **h**. IPGTT conducted 30 minutes after IP injection of 0.008 nmol/kg of Ex-4 to 12–14-week chow fed *Gpt2*^f/f^ or *Gpt2*^βKO^ mice**. i**. Intraperitoneal GSIS. Insulin levels assessed during the IP Ex-4 administration. N=8–10/group, Mean ± SEM, significance calculated by 2-way ANOVA and Tukey’s multiple comparisons. *P<0.05, **P<0.01.

We previously identified mitochondrial transaminase *Gpt2* as a transcriptional target of a stress-inducible PDX1-ATF transcriptional complex. Stress upregulation and the presence of a conserved PDX-1 binding CARE motif near the human *GPT2* locus suggested conservation of this regulatory network across species. Moreover, silencing *Gpt2* in primary mouse islets exposed to thapsigargin or glucolipotoxic stress markedly reduced β-cell apoptosis^22,29^. Here, we set out to investigate the physiological repercussions of Gpt2 depletion in pancreatic β-cells *in vivo*. We generated a *Gpt2* deficiency mouse model (*Gpt2*^βKO^), with *Gpt2*^f/f^ mice as controls (Fig.1a). Efficient deletion was verified by western blot analysis (Fig.1b, c). A mild improvement in glucose tolerance and insulin secretion in response to intraperitoneal glucose challenge was observed in *Gpt2*^βKO^ mice compared to *Gpt2*^f/f^ littermates (Fig1d and 1e, respectively). In contrast, when challenged with oral glucose, *Gpt2*^βKO^ mice had significantly improved glucose tolerance and higher insulin secretion than control littermates (Fig.1f, g). Minimal phenotypes were observed in female *Gpt2*^βKO^ mice (Supplementary Fig.1).

The preferentially higher response of *Gpt2*^βKO^ mice to oral glucose administration suggested an enhanced incretin effect. GLP1 and GIP, the two major incretin hormones, account for 50–70% of insulin secreted in healthy individuals after meal intake. GLP-1 and GIP have also been implicated in the suppression of β-cell apoptosis, increased β-cell mass and the amelioration of glucotoxic effects during diabetes^30–32^, and loss of incretin sensitivity, a hallmark of type-2 diabetes^12^. To assess beta cell sensitivity to incretin hormones, we injected a low dose of the dipeptidyl peptidase 4 (DPP4) resistant GLP1R agonist, Exendin-4 (Ex4) (0.008ng/kg body weight)^33,34^ to male *Gpt2*^f/f^ and *Gpt2*^βKO^ mice and performed an intraperitoneal glucose tolerance test (IPGTT) 30 mins later. Following Ex4, *Gpt2*^βKO^ mice showed greater initial reduction in blood glucose as well as lower blood glucose excursion compared to *Gpt2*^f/f^ littermates (Fig.1h). Concomitantly, *Gpt2*^βKO^ mice also secreted significantly more insulin at 30-, 45-, and 60-minutes after Ex4, suggesting a heightened β-cell response to the GLP-1R agonist (Fig.1i).

### Improved incretin sensitivity of *Gpt2*^βKO^ mouse islets

**Figure 2.**
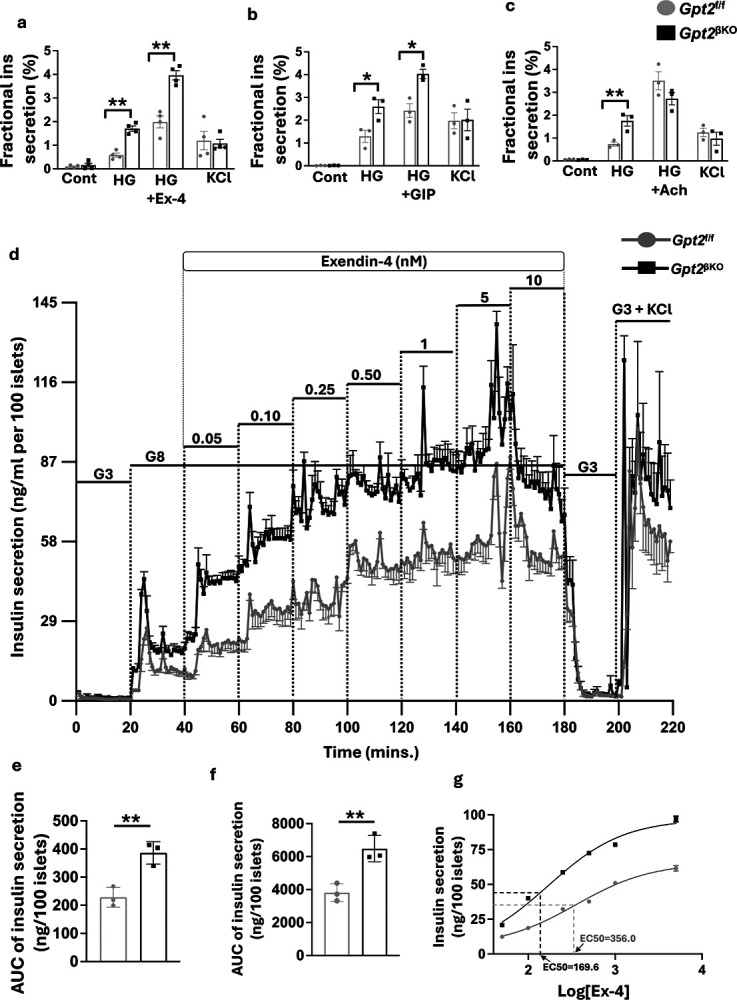
β-cell *Gpt2* deficiency improves islet GLP1 and GIP sensitivity. GSIS in batch incubations of *Gpt2* ^f/f^ and *Gpt2*^βKO^ mouse islets incubated with basal (2.8 mM), high glucose (25mM), KCl (30mM) and **a**. Ex4 (1nM); **b**. GIP (1nM); **c**. Acetylcholine (Ach, 0.5 μM). Data presented as fractional insulin secretion (%). Mean± SE, n=3–4. Significance calculated using 2-way ANOVA with Tukey’s multiple comparisons (*P< 0.05, **P < 0.01). **d**. Dynamic insulin secretion profile of isolated *Gpt2*^f/f^ or *Gpt2*^βKO^ islets in response to low glucose (G3, 3mM glucose), high glucose (G8, 8mM glucose), stepwise increases of Ex4 (50pM to 10nM), stimulus washout in G3, and final stimulation with G3+30mM KCl (N=3 biological replicates/genotype).. Area under the curve (AUC) showing differences in responses during **e**. G8, and **f**. G8 and Ex-4 (0.05–5 nM) steps of the perifusion assay. **g**. Dose-response curve showing the relation between Exendin-4 concentration and insulin secreted from *Gpt2*^f/f^ and *Gpt2*^βKO^ islets. EC50 value determined using non-linear curve fitting of the dose response curve.

In addition to the well-established direct effects on pancreatic islet β-cells, GLP1R agonists exert pleiotropic effects on peripheral tissues, including liver, brain and gut, which can in turn influence β-cell function. Therefore, we investigated if our *in vivo* observations could be recapitulated in isolated islets under *ex vivo* conditions. Isolated *Gpt2*^f/f^ control and *Gpt2*^βKO^ islets were incubated with basal glucose (2.8mM), followed by high glucose (30mM), and then high glucose with one of 3 different insulin secretogogues: Ex4 (1nM), GIP (1nM), or Acetylcholine (Ach; 0.5uM), and finally with KCl (30mM KCl in 2.8mM glucose) (Fig.2a,b, and c respectively). Isolated islets from *Gpt2*^βKO^ mice had higher insulin secretion in response to high glucose in all three cases, indicating that β-cell depletion of *Gpt2* enhances glucose responsiveness. Interestingly, *Gpt2*^βKO^ islets had markedly higher insulin secretion in response to Ex4 and GIP, but not acetylcholine. To ascertain if *Gpt2* depletion enhances islet sensitivity to incretin hormones, we conducted a dynamic perifusion assay wherein *Gpt2*^f/f^ and *Gpt2*^βKO^ islets were exposed to low glucose (3mM), high glucose (8mM), and stepwise increments of Ex4 (50pM −10nM) in the presence of 8mM glucose (Fig.2d). *Gpt2*^βKO^ islets had a higher response to 8mM glucose and markedly enhanced insulin secretion during stepwise addition of Ex4 and a lower EC50 (169.6 nM for *Gpt2*^βKO^ islets vs 356 nM for *Gpt2*^f/f^ islets), indicating greater sensitivity to Ex4 (Fig. 2e–g). Taken together, these results indicate that the augmented incretin responsiveness observed in *Gpt2*^βKO^ mice represents an islet-autonomous phenomenon in which *Gpt2* depleted β-cells are more sensitive to the GLP-1R agonist Ex4.

### Enhanced incretin effect in *Gpt2*^βKO^ mice during HFD-feeding

**Figure 3.**
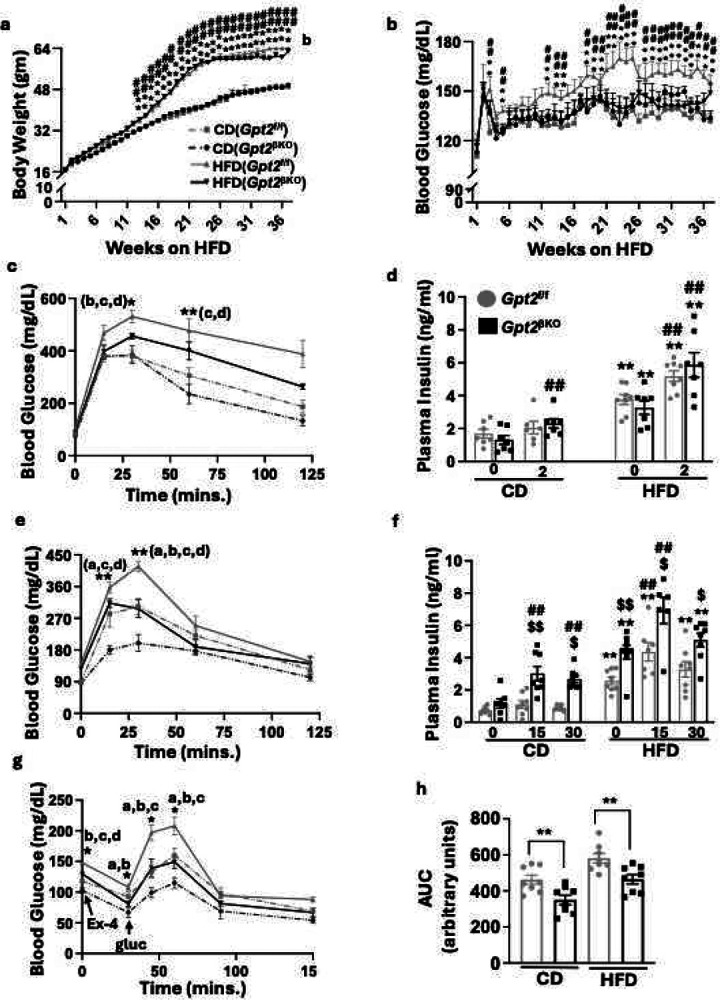
Augmented incretin effect during prolonged HFD feeding of *Gpt2*^βKO^ mice. *Gpt2*^f/f^ or *Gpt2*^βKO^ mice fed either 45 kcal% fat HFD or matched 10 kcal% fat control diet (CD) for 37 weeks, and *ad libitum* **a**. body weight and **b**. blood glucose were recorded weekly. Values are means ± SEM. Significance calculated using 2-way ANOVA and Tukey’s multiple comparisons. *P<0.05, **P<0.01**P<0.05 : *Gpt2*^f/f^ HFD vs *Gpt2* ^f/f^ CD; ^##^ P< 0.05: *Gpt2*^βKO^ HFD vs *Gpt2*^βKO^ CD. N=7–8/group. **c**. IPGTT performed on *Gpt2*^f/f^ or *Gpt2*^βKO^ mice after 14 weeks on HFD or CD. and **d**. IPGSIS performed on *Gpt2*^f/f^ or *Gpt2*^βKO^ mice after 26 wk on HFD or CD. **e**. OGTT oral assessed on *Gpt2*^f/f^ or *Gpt2*^βKO^ mice after 15wk HFD or CD feeding. **f**. Oral GSIS after 17 weeks on HFD or CD. For IP and oral GTT: values are mean ± SE, *P<0.05, **P<0.01 for the following groups: ^a^*Gpt2*^f/f^ CD vs *Gpt2*^βKO^ CD, ^b^*Gpt2*^f/f^ HFD vs *Gpt*2^βKO^ HFD, ^c^*Gpt2*^f/f^ HFD vs *Gpt2*^f/f^ CD, ^d^*Gpt2*^βKO^ HFD vs *Gpt2*^βKO^ CD; N=5–8 per group. For IP and oral GSIS : values are mean ± SE. Two-way ANOVA, followed by Tukey’s multiple comparison test. *, P < 0.05, **, P < 0.005 relative to corresponding Control Diet. #, P < 0.05, ##, P < 0.005 relatives to the 0-time point in the respective diet group. $, P < 0.05, $$, P < 0.005 relative to diet matched *Gpt2*^f/f^ littermates. N=5–8/group. **g**. IPGTT conducted 30 minutes after IP injection of 0.01 nmoles/kg Ex4 to *Gpt2*^f/f^ or *Gpt2*^βKO^ mice after 23 weeks on HFD or CD. N=7–8/group. Means ± SEM, 2 way ANOVA and Tukey’s multiple comparisons. *P<0.05 for the following groups: ^a^*Gpt2*^f/f^ CD vs *Gpt2*^βKO^ CD, ^b^*Gpt2*^f/f^ HFD vs *Gpt2*^βKO^ HFD, ^c^*Gpt2*^f/f^ HFD vs *Gpt2*^f/f^ CD, ^d^*Gpt2*^βKO^ HFD vs *Gpt2*^βKO^ CD. **h**. AUC quantification of blood glucose levels over time rafter glucose injection at 30 minutes post Ex-4 in **g**.

Loss of β-cell incretin sensitivity is a hallmark of T2D, contributing to impaired glucose homeostasis^4,35^. In mice, metabolic stress induced by high calorie diet has been reported to mimic the diabetic milieu and negatively impact the incretin response by lowering β-cell sensitivity to incretin hormones^36^. We, therefore assessed the impact of prolonged high fat (HFD, 45 kcal%) or matched control diet (CD) feeding on *Gpt2*^βKO^ and *Gpt2*^*f*/f^ mice. During the 37 weeks of diet intervention, the *Gpt2*^βKO^-HFD group showed markedly lower non-fasting, weekly blood glucose levels than the *Gpt2*^*f*/f^-HFD group (Fig.3b), despite similar weight gain (Fig.3a). Insulin tolerance was unchanged between the genotypes on either CD or HFD, indicating that changes in glucose tolerance are likely due to improvement in β-cell function (Supplementary Fig.2c). Surprisingly, assessment of the glycemic profile following intraperitoneal injection of glucose showed only mild improvement in glucose tolerance (Fig.3c) and no improvement in insulin secretion (Fig.3d). In striking contrast, following oral glucose injection, the *Gpt2*^βKO^ mice showed marked improvements in both glucose tolerance (Fig.3e) and insulin secretion (Fig.3f). Circulating levels of the two major incretin hormones, GLP-1 and GIP, were unaltered (Supplementary Fig. 2a and b, respectively), suggesting that enhanced incretin sensitivity of *Gpt2* depleted β-cells is responsible for the improved β-cell function. To specifically examine the effect of *Gpt2* in modulating β-cell incretin sensitivity, we injected *Gpt2*^βKO^ and *Gpt2*^*f*/f^ littermates with Ex4 and performed an IPGTT 30 minutes later. *Gpt2*^βKO^ mice showed an improved glycemic profile and markedly higher insulin secretion after Ex4, consistent with our previous observations in chow fed mice (Fig. 3g and 3h). Thus, depletion of *Gpt2* in β-cells enhances incretin sensitivity of HFD fed mice, leading to improved glucose homeostasis.

### β-cell *Gpt2* deficiency enhances the pro-survival effect of Ex4

**Figure 4.**
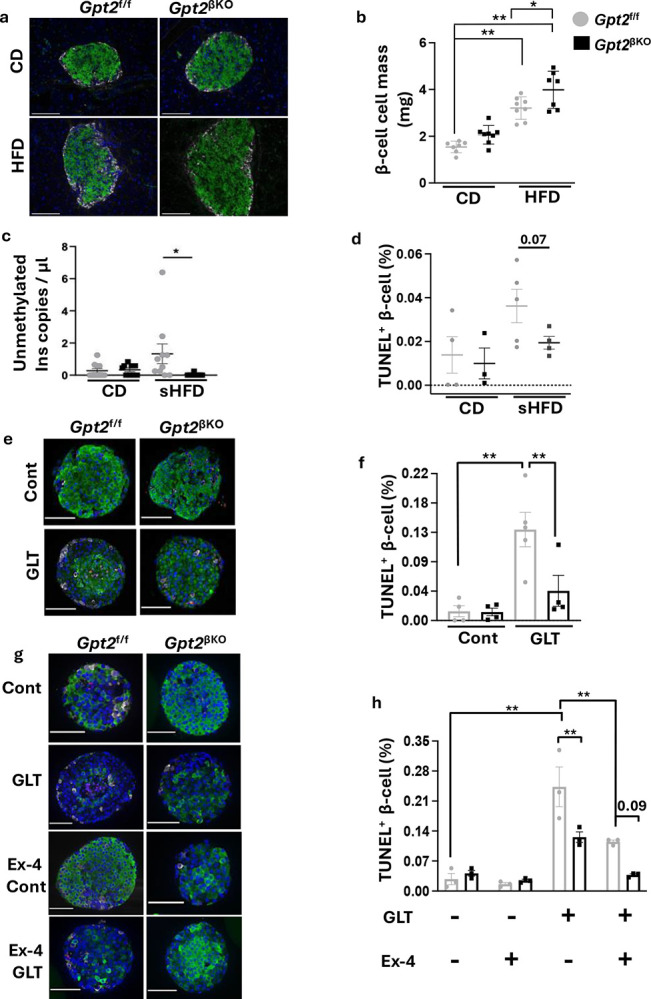
β-cell *Gpt2* deficiency enhances the pro-survival effect of Ex4. **a**. Immunofluorescence staining (Hoechst(blue), insulin (green), and glucagon (grey)). Scale bar, 50μm. **b**. Quantification of β cell mass of *Gpt2*^f/f^ and *Gpt2*^βKO^ mice after 37 weeks on HFD or CD. Mean±SE. N=7–8/ group. P value calculated by two-way ANOVA, followed by Tukey’s multiple comparisons. *, P<0.05. **, P < 0.01. **c**. ddPCR quantification of unmethylated Ins DNA from serum of *Gpt2*^f/f^ or *Gpt2*^βKO^ mice at the end of 1 week HFD or CD feeding, N= 9–11/group. *P<0.05 by ordinary one-way ANOVA, followed by Tukey’s multiple comparisons. **d**. Quantification of TUNEL+ insulin+ cells from pancreatic sections of *Gpt2*^f/f^ or *Gpt2*^βKO^ mice at the end of 1 week HFD or CD feeding. Mean+SE. N=4–6/group. P value calculated by one-way ANOVA, followed by Tukey’s multiple comparisons. **e**. Representative immunofluorescence imaging of isolated islets from *Gpt2*^*f*/f^ and *Gpt2*^βKO^ mice exposed to three days of control or GLT conditions. Islets fixed, sectioned, and stained for Hoechst (blue), insulin (green), glucagon (grey), and TUNEL (red). Scale bar, 50μm. **f**. β-cell death expressed as a percentage of TUNEL^+^ insulin^+^ cells. Mean± SEM, N=3/ group. ≥1000–1500 insulin^+^ cells counted per group. **P<0.01 by ordinary one-way ANOVA, followed by Tukey’s multiple comparison test. **g**. Representative immunofluorescence image of isolated islets from *Gpt2*^f/f^ and *Gpt2*^βKO^ mice exposed to three days of control or GLT condition, with or without 10nM Ex4 starting 24 hrs prior to GLT. Islets fixed, sectioned, and stained for Hoechst (blue), insulin (green), glucagon(grey), and TUNEL (red). Scale bar, 50μm. **h**. quantification of **g**. β-cell death expressed as a percentage of TUNEL^+^ insulin^+^ cells. N=3/group; ≥1000–1200 insulin^+^ cells counted per group. **P<0.01 by two-way ANOVA, followed by Tukey’s multiple comparison test.

β-cell mass was increased in *Gpt2*^βKO^ mice compared to littermate control mice after long term HFD feeding (Fig. 4a, b). The number of TUNEL^+^ β-cells at end of the study was not elevated; however, HFD-induced β-cell death is episodic, and apoptotic cells are rapidly cleared, thus limiting detection^37–39^. Postnatal β-cell mass expansion in rodents occurs by replication of pre-existing β-cells^40,41^. Hence, to determine whether enhanced survival of β-cells early during HFD feeding preserves a pool of functional β-cells allowing for later compensatory expansion of β-cell mass, we assessed β-cell death *in vivo* in 5-week-old mice after 7 days of HFD-feeding by digital droplet PCR of circulating unmethylated copies of insulin DNA and by TUNEL staining of pancreatic sections at the end of the diet intervention. After 1-week HFD feeding, elevated circulating unmethylated copies of *Ins* DNA (Fig. 4c) and TUNEL^+^ β-cells (Fig. 4d) were observed in *Gpt2*^f/f^ mice, and these effects were mitigated in *Gpt2*^βKO^ mice, indicating a protection from metabolic stress induced β cell death conferred by β cell *Gpt2* deficiency.

The protection of β-cell survival by *Gpt2* deficiency was recapitulated *ex vivo*. Isolated *Gpt2*^f/f^ or *Gpt2*^βKO^ islets were maintained under control or GLT conditions for 3 days. *Gpt2* deletion attenuated beta cell death during GLT, evidenced by a more than three-fold reduction in the percentage of TUNEL^+^ β-cells (Fig. 4e, f). Finally, we assessed the impact of Ex4 on GLT stress-induced apoptosis of *Gpt2*^f/f^ and *Gpt2*^βKO^ β-cells. Ex4 lowered TUNEL^+^ β-cells in *Gpt2*^f/f^ islets, pointing to a protective effect rendered by incretin activation during GLT stress. Remarkably, the combination of *Gpt2* deficiency and Ex4 lowered death to control levels, strongly suggesting that *Gpt2* silencing augments the pro-survival action of Ex4 during GLT stress (Fig. 4g and 4h).

### β-cell *Gpt2* silencing counters the transcriptional effects of glucolipotoxicity

**Fig. 5:**
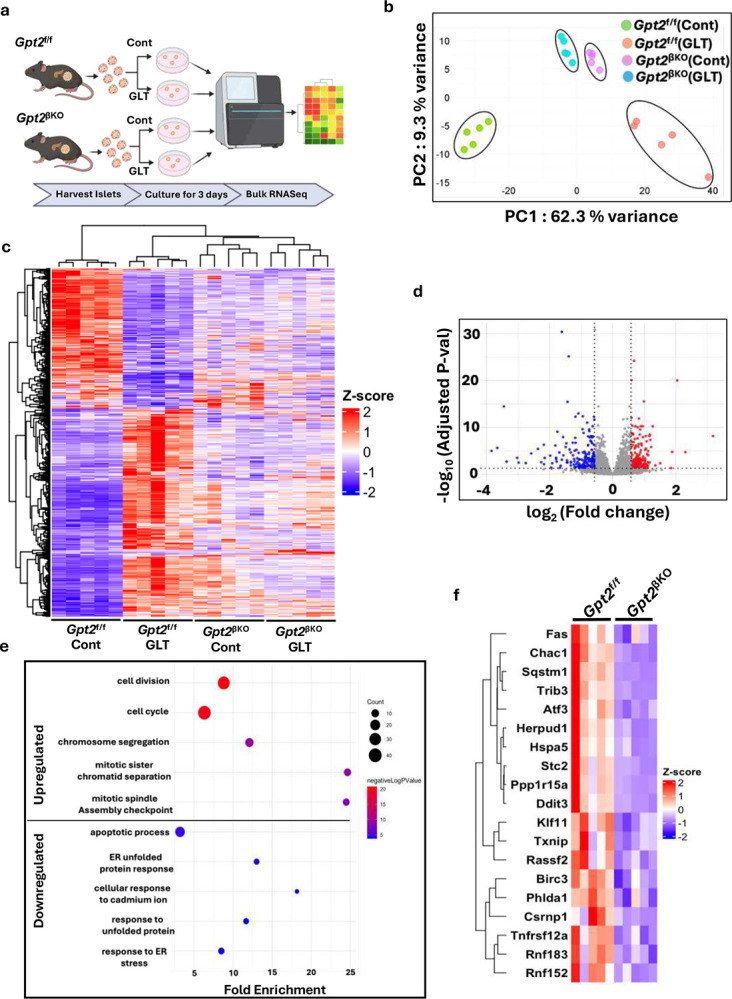
β-cell *Gpt2* silencing promotes a pro-survival transcriptional program during GLT stress. **a**. Schematic of RNA-seq experimental design generated on BioRender N=5 biological replicates/group, Cont= 11mM glucose, GLT = 25mM glucose + 0.5mM free fatty acid. **b**. PCA plot **c**. Heatmap showing hierarchical clustering of differentially expressed genes (DEG) (adjusted P-value ≤ 0.05, log2 fold change ≥ 0.58).. **d**. Volcano plot showing upregulated (red) and downregulated (blue) genes from GLT exposed *Gpt2*^βKO^ and *Gpt2*^f/f^ islets. Genes with no significant change (grey). **e**. Top 5 GO biological process terms obtained from DAVID enrichment analysis of upregulated or downregulated DEGs in GLT exposed *Gpt2*^βKO^ and *Gpt2*^f/f^ islets. **f**. Clustered heatmap showing relative change in expression of stress response and apoptosis related genes in GLT exposed *Gpt2*^βKO^ and *Gpt2*^f/f^ islets.

To understand the global impact of *Gpt2* depletion on biological processes and pathways, we performed bulk RNAseq analysis of *Gpt2*^f/f^ and *Gpt2*^βKO^ islets cultured under control or GLT conditions for 3 days (Fig.5a). We observed a significant stratification by genotype and culture condition, indicating substantial differences in gene expression profiles and underlying biological pathways among these conditions (Fig. 5b). Expression levels of significantly altered genes are depicted as hierarchical clustering in a heatmap across sample groups (Fig.5c).

β-cell damage in patients with T2D is often attributed to elevated levels of circulating glucose and lipid^42,43^; hence, multiple studies have used the *ex vivo* GLT model in islet cultures to replicate the metabolic milieu of T2D^16,44^. To understand how closely *ex vivo* GLT stress recapitulates T2D, we compared the impact of GLT on gene expression of control (*Gpt2*^f/f^) islets to two publicly available bulk RNAseq datasets of human islets from non-diabetic donors or donors with T2D: (T2D_1^45^ and T2D_2^46^) We also compared these data with publicly available bulk RNAseq data of *db/db* mouse islets^47^ which phenocopy the β-cell dysfunction prevalent in T2D islets^48^. We observed significant overlap with gene expression of T2D and *db/db* islets, indicating that GLT exposed *Gpt2*^f/f^ islets show congruency in the transcriptional landscape and thus similarity in underlying molecular mechanisms with pathophysiologically relevant β-cell models (Supplementary Fig. 3a).

We next focused on differential gene expression between GLT-treated Gpt2^βKO^ and Gpt2^f/f^ islets to assess how *Gpt2* silencing influences the islet response to GLT stress. We found 352 upregulated and 442 downregulated genes (Fig. 5d). The five most significant biological processes enriched in the downregulated gene group were related to ER-stress and apoptosis, whereas the up-regulated gene group highlighted pro-survival programs, such as cell-division and cell-cycle regulation (Fig. 5e). Heatmaps of a key set of genes from ER-stress and apoptosis (Fig. 5f) and from cell-cycle and cell-division pathways (Supplementary Fig. 3b) are shown. We also observed upregulation of key cAMP pathway factors (*Crem*, *Irs2*) along with cell-cycle and IGF related pathways in *Gpt2*^βKO^ versus *Gpt2*^f/f^ islets under control conditions (Supplementary Fig.3c and 3d). *Gpt2* silencing under GLT conditions mirrored previously reported transcriptional effects of Ex4, including lower expression of the ER-stress and pro-apoptotic genes *Fas*^49^ and *Ddit3*^50^ and upregulation of the proliferative gene *Ccna2*^51^. Together, these transcriptional changes support our physiological observations that *Gpt2* depletion primes β-cells for enhanced incretin signaling while simultaneously activating intrinsic survival pathways.

### GPT2 silencing lowers apoptosis and restores incretin responsiveness of human T2D β-cells

**Figure 6.**
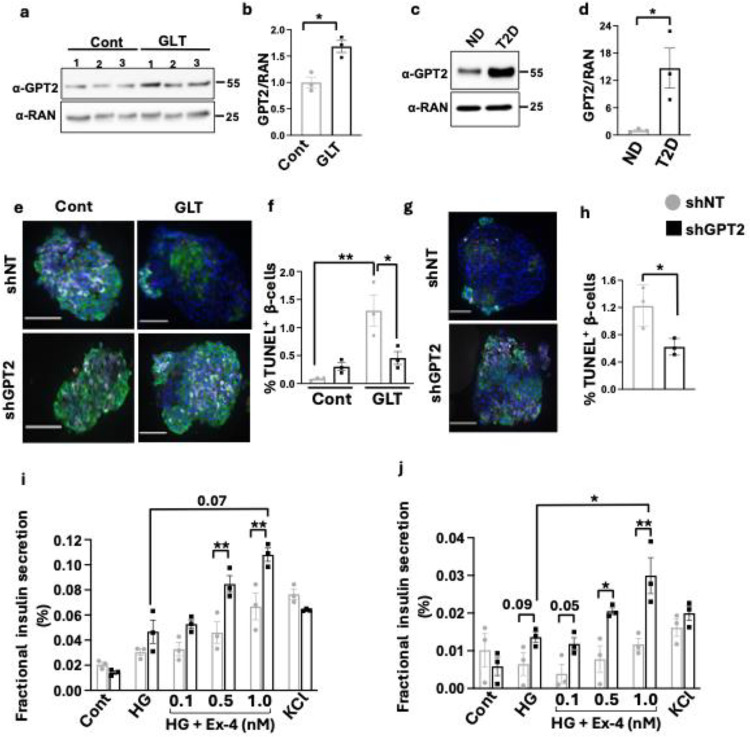
GPT2 silencing lowers apoptosis and restores incretin responsiveness of human T2D β-cells. a. Western blot (WB) analysis of human islets obtained from 3 independent non-diabetic donors maintained under control (Cont, 11mM glucose), or GLT (25mM glucose + 0.5 mM free fatty acid) conditions for 3 days. b. Densitometric data normalized to RAN (right). *P<0.05, by unpaired two tailed t test. c. Representative WB of GPT2 in islets from non-diabetic (ND) donors and donors with type 2 diabetes (T2D). d. Densitometric quantification of c. normalized to RAN. *P<0.05, by unpaired two tailed t test. e. Representative image of non-diabetic human donor islets transduced lentivirally with either shNT or sh*GPT2*, followed by 3 days of culture in control or GLT conditions. Islets were fixed, sectioned, and stained for Hoechst (blue), insulin (green), glucagon (grey), and TUNEL (red). f. β-cell apoptosis quantified from data in e. Percentage of TUNEL+Ins+ cells. *P < 0.05, **p<0.01, 2-way ANOVA followed by Tukey’s multiple comparison test. g. Representative image of human islets from a donor with T2D transduced lentivirally with either shNT or sh*GPT2*, and stained for Hoechst (blue), insulin (green), glucagon (grey), and TUNEL (red). h. β-cell apoptosis quantified from g. Percentage of TUNEL+Ins+ cells. *P < 0.05, **p<0.01, 2-way ANOVA followed by Tukey’s multiple comparison. ≥1500 Ins+ cells quantified. Scale bar, 30 μm. Static incubation of shNT or sh*GPT2* transduced human islets from a donor i. without diabetes or j. with T2D incubated for 45 minutes in the indicated conditions. Islets from each donor, transduced with shNT or sh*GPT2*, were divided into three groups of 30 islets each and each group treated as a technical replicate. Data presented as mean ± SE. Significance calculated using 2-way ANOVA with Tukey’s multiple comparisons (*P< 0.05, **P < 0.01).

To establish the potential translational relevance of these findings to human disease, we analyzed human islets from non-diabetic donors and from donors with T2D. Similar to mouse islets, GPT2 was induced by GLT in human islets (Fig.6a, b). GPT2 was also markedly increased in islets from T2D donors (Fig.6c, d, Supplementary Fig. 4a). To determine whether *GPT2* deletion protects human β-cells from GLT stress-induced apoptosis, we transduced islets from non-diabetic donors with lentivirus directing human β-cell expression of shNT or shGPT2 and exposed them to control or GLT stress conditions. In the transduced islets, 66–88 % insulin-positive β-cells were EGFP positive, confirming high β-cell-specificity for both shRNA constructs (Supplementary Fig. 5c). We observed a significant increase in apoptotic β-cells under GLT stress in these islets, and this was remarkably counteracted by depletion of *GPT2* (Fig.6e–f). Importantly, *GPT2* silencing in the β-cells of islets from T2D donors significantly reduced the number of apoptotic β-cells (Fig.6g–h).

Incretin responsiveness is impaired in islets from patients with T2D^5,52,53^. To determine the impact of *GPT2* silencing on incretin sensitivity of human β-cells, islets from a single, non-diabetic donor were again transduced with shNT or shGPT2 lentivirus, and insulin secretion was assessed after static incubation with basal glucose (2.8mM), high glucose (8mM), and increasing doses of Ex4 in the setting of high glucose (Fig. 6i). To ensure that transduction itself did not alter glucose and Ex4 responsiveness of donor islets, we performed static incubation with un-transduced non-diabetic islets under the same conditions and observed no difference with shNT-transduced islets (Supplementary Fig.4e). GPT2 silencing enhanced Ex4 stimulated insulin secretion, suggesting increased GLP1 sensitivity on reduction of *GPT2*. We further tested the incretin responsiveness of type 2 diabetic islets after *GPT2* silencing and observed improved secretion in response to high glucose and enhancement of Ex4 stimulated insulin secretion, suggesting that GPT2 deletion can restore incretin sensitivity and beta cell function in islets from T2D donors (Fig. 6j).

## Discussion

Pancreatic β-cells integrate metabolic and hormonal signals to regulate insulin output^54–56^. Incretin hormones are critical not just for regulating insulin secretion, but also for the survival and replication of β-cells^2,3,57,58^. Despite inherent differences in modes of β-cell incretin activity between murine and human models^59,60^, we present GPT2 as a key factor regulating β-cell incretin activity and survival across species. *Gpt2*^βKO^ mice exhibited improved incretin sensitivity and enhanced β-cell mass expansion under HFD due to improved β-cell survival. These findings were corroborated in human islets as well, where GPT2 was induced in the context of T2D and in non-diabetic islets exposed to GLT conditions

This work thus establishes a link between glutamine metabolism and incretin stimulated insulin secretion in the beta cell. Glutamine metabolism plays an important role in insulin secretion^61,62^, and it has been suggested, based on experiments in β-cell lines, that glutamate transport into secretory vesicles promotes insulin granule recruitment to the plasma membrane and is thus linked to incretin-stimulated insulin secretion^63^; however, based on the pattern of KCl-stimulated insulin secretion during our GSIS assays, *Gpt2* appears to affect mechanisms further upstream. This is additionally supported by the lack of impact of β-cell *Gpt2* deficiency on Ach-stimulated secretion. Further investigation of the intersection of glutamine metabolism with incretin hormone signaling in the β cell and the relationship of these pathways to insulin secretion and cell survival is warranted.

*Gpt2* silencing in β-cells also amplifies the pro-survival effect of the GLP1R agonist Ex4. Previous studies point towards pro-proliferative and anti-apoptotic roles of incretin signaling in β-cells, linked to upregulation of genes like *Ccna2*^51^ and *Irs2*^64,65^ and to downregulation of *Fas* and *Ddit3*. Our transcriptomic analysis revealed upregulation of *Ccna2* and downregulation of *Fas* and *Ddit3* by *Gpt2* silencing during GLT stress whereas key effectors of GLP1R signaling in β-cells like *Crem*^66,67^ and *Irs2* were upregulated by *Gpt2* silencing under control conditions, altogether demonstrating that *Gpt2* silencing counters the effects of glucolipotoxicity and providing pathways through which *Gpt2* silencing promotes Ex4 effects on β cell function and survival.

A growing body of studies on incretins highlights their dual benefit to enhance insulin secretion and preserve β-cell mass. Our study suggests that selective targeting of GPT2 could amplify the therapeutic benefit of GLP-1R agonists to optimize glycemic control. Thus, our findings position GPT2 as a promising therapeutic target for enhancing β-cell lifespan and function during diabetes.

## Methods

### Generation of conditional *Gpt2* deficiency in mice

To generate β-cell specific deletion of *Gpt2* in mice (*Gpt2*^βKO^), we intercrossed C57BL/6 mice with loxP sites flanking exon 4 of *Gpt2* (*Gpt2*^f/f^)^26^ with a β cell specific Cre recombinase deleter strain (Tg(*Ins2-Cre*)^Herr^)^68^. This deleter strain is characterized by high efficiency targeting of β cells, absence of a GH expressing mini-cassette, minimal brain leakinesss and long history of effective targeting without silencing^69^. We previously documented normal glucose homeostasis in male and female Tg(*Ins2-Cre*)^Herr^ mice^70,71^. *Gpt2*^f/f^ littermates served as controls. All animals were housed in the animal care facility of the University of Pennsylvania and maintained on a 12-hour light/dark cycle, with *ad libitum* access to food and water. Unless otherwise indicated, experiments were performed on male mice.

### *In vivo* metabolic phenotyping

Mice were fasted overnight before administration of 2g/kg BW glucose either by intraperitoneal injection or by oral gavage for glucose tolerance testing (GTT) as previously described^70–72^. Exendin-4 (Ex4; Millipore Sigma, E7144) was reconstituted in sterile 0.9% NaCl, 1% BSA and injected intraperitoneally. Intraperitoneal GTT or glucose stimulated insulin secretion (GSIS) was assessed 30 minutes later. Insulin tolerance testing (ITT) was performed by injecting 0.75U/kg BW insulin (Novolin, 0169–18341) after a 6 hour fast. For both IP- and OGSIS, 3g/kg BW glucose was injected either intraperitoneally or administered by oral gavage. For glucose and insulin measurements, tail vein blood was collected into EDTA coated collection vials (Sarstedt, 16.444.100) followed by plasma separation by centrifugation and storage at −80°C. Insulin was assayed by insulin ELISA (Crystal Chem, 90082) following manufacturers protocol. For measurement of GLP-1 and GIP, *Gpt2*^f/f^ and *Gpt2*^βKO^ mice were administered 3g/kg BW glucose by oral gavage and plasma collected from the tail vein was separated as described above. GLP-1and GIP were assessed by ELISA (Meso Scale diagnostics, K1503PD-1 and Crystal Chem, 81527).

For high fat diet (HFD) feeding, 4-week-old male *Gpt2*^f/f^ and *Gpt2*^βKO^ mice were weaned onto either a HFD (45% Kcal fat, Research Diets: D12451) or matched control diet (10% Kcal fat, Research Diets: D12450H) for 1 or 37 weeks. Weekly, non-fasting body weight and blood glucose were measured. Blood glucose was measured from a nick in the tail vein by handheld glucometer.

### Isolation and culture of human and mouse pancreatic islets.

Primary mouse islets were isolated as previously described^70^ and recovered for 3 days in RPMI 1640 medium supplemented with 11 mM glucose and 10% FBS. De-identified human islets from non-diabetic donors or donors with T2D were obtained through the Integrated Islet Distribution Program or the Human Pancreas Analysis Program, both NIH-approved centers with informed consent and IRB approval. Donor islet characteristics are provided in Supplementary Table 4. For glucolipotoxicity experiments, mouse and non-diabetic human islets were incubated with either control (RPMI,1% BSA) or GLT media containing RPMI supplemented to 25mM glucose and with 0.5 mM FFA (2:1 mix of palmitate and oleate) and 1% BSA for 3 days after recovery^73^.

### Cell culture and shRNA preparation

HEK293T cells were cultured in DMEM (Gibco, 11965084) with 10% FBS (GeminiBio, S11150) and 1% penicillin-streptomycin (Thermo Fischer, 15140122). For lipotoxic stress treatment, cells were incubated in the indicated concentrations of FFA with 1% BSA in culture media, with 1% BSA serving as control.

For shRNA mediated silencing in human islets, shRNA sequences targeting the human *GPT2* transcript (ENST00000340124.9) were designed using the Sherwood shRNA design algorithm^74^ and 3 shRNAs targeting different regions of the *GPT2* transcript were individually cloned into a pLenti plasmid vector that contains CMV promoter driving expression of EGFP with an shRNA acceptor sequence in its 3’UTR^70^. Plasmids were transfected into HEK293T cells using lipofectamine 2000 to assess silencing efficiency.

To create a human β-cell specific shRNA expression system, we first used Gibson-assembly to introduce a RIP1-miniCMV enhancer-promoter that confers >90% high human β cell specificity^75^ into our pLenti EGFP construct. The *GPT2* shRNA construct showing highest silencing efficiency in HEK293T cells was cloned into the 3’UTR shRNA acceptor cassette downstream of the EGFP coding sequence and packaged into lentiviral particles. Lentiviral packaging vectors (psPax2 and PMD2G) were transfected to HEK293 cells together with our pLenti-shRNA constructs. Lentiviral particles in the culture media were collected at 24-, 48-, and 72-hours using Lenti-X Concentrator (631232, Takara Biosciences) following manufacturer protocol and resuspended in culture medium before being aliquoted and stored in −80°C. Human islets were transduced with lentivirus media overnight in serum-free media as described previously. To assess β-cell-specificity, paraffin sections of donor islets from non-diabetic individuals transduced with non-targeting (shNT) or *GPT2* (shGPT2) shRNAs were stained with anti-insulin, -glucagon or -EGFP antisera. Sequence of the shRNAs tested will be made available upon request.

### Immunohistochemistry and Immunofluorescence

Harvested pancreata were weighed before overnight fixation in 4% paraformaldehyde at 4°C followed by paraffin embedding and sectioning. For β-cell mass measurement, 3 sections per animal taken at ~ 200μm intervals in the region of maximal footprint were stained with guinea pig anti-insulin antiserum (Dako), mouse anti-glucagon antiserum (Millipore) and counterstained with Hoechst (Sigma). Antigen retrieval was performed using 20ug/ml Proteinase K in 10mM Tris-HCl, pH-8.

Mouse and human islets were fixed in 4% paraformaldehyde for 30 minutes at room temperature, followed by resuspension in warm histogel and shaping into small discs. Histogel discs were paraffin embedded and sectioned as described previously^76^. Paraffin slides were treated with 10mM sodium citric acid buffer pH 6 with 0.2% Tween20 in a pressure cooker for 2 hours and stained with primary antisera to goat anti-GFP, insulin and glucagon. For β-cell specific shRNA expression in human islets, EGFP expression was used as marker for transduction efficiency and specificity. To assess β-cell death, the In-situ Cell Death Detection kit (Fluorescein; Millipore Sigma, 11684795910) was used following manufacturer’s instructions along with fluorescent conjugated dUTP (Invitrogen A32762). TUNEL+ β-cell number was normalized to total β-cell number.

Images were captured on a QImaging Q/Click digital camera equipped Nikon Eclipse E600 microscope using MetaMorph software. For whole slide scanning at 20X magnification, we used an Aperio Versa 200 slide scanner and images were analyzed using Aperio Imagescope software (Leica biosystems). For each animal, the fraction of total insulin-positive area/total footprint area was multiplied by the respective pancreas wet weight to determine beta cell mass.

For isolated islet sections, scanning was performed with an Agilent Biotek Cytation5 Imaging reader using a 20X/0.45NA Plan-Fluorite phase objective. Captured images were analyzed using Qupath image analysis software (version 0.5.1)^77,78^. Details of primary and secondary antisera used for immunofluorescence are provided in Supplementary Table 3.

### Static and dynamic insulin secretion assays

For static incubation studies using mouse or human islets, 25–30 islets were washed and incubated in Krebs-Ringer bicarbonate buffer (KRBB), supplemented with the indicated concentrations of glucose, Ex4, GIP (Fisher Scientific, NC9796977), Acetylcholine (Millipore Sigma, A2661), and potassium chloride (KCl) for 45 minutes each as previously described^70,71^. At the end of the experiment, the islets were extracted for later measurement of insulin content. Insulin content and insulin secreted into the medium for each condition was measured by insulin ELISA (Crystal Chem, for mouse (90082) and human (90115) insulin). Secreted insulin was normalized by insulin content to calculate fractional insulin secretion.

Dynamic perifusion assays were carried out with a Biorep Perifusion PER-04 device. One hundred islets from *Gpt2*^f/f^ or *Gpt2*^βKO^ were perifused at 100μl/min with low glucose (G3, 3mM), high glucose (G8, 8mM), followed by stepwise increments of Ex4 (50pM-10nM) in G8 and followed by washout in low glucose and 30mM KCl. Perifusates were collected every minute and assayed in triplicate for insulin by the Radioimmunoassay and Biomarker Core of the Penn Diabetes Research Center.

### Digital droplet PCR (ddPCR) for unmethylated Ins DNA

At the end of the 1-week HFD study, blood was collected by cardiac puncture from mice under isoflurane inhalation anesthesia (1.5% v/v O_2_), and serum obtained after centrifugation was stored at −80°C. ddPCR for quantification of differentially methylated Ins gene promoter DNA was carried out as described^79^.

### RNA sequencing and data analysis

At least 150 islets per genotype and condition were resuspended in TRIzol, RNA was isolated, and sequencing libraries were prepared using the Illumina Ultra Low Input RNA kit. Libraries were then sequenced on a HiSeq 2500 system with a 150-nt paired-end read protocol. Reads from the FASTQ files were processed by CutAdapt 1.15 to detect and trim adapter sequences. Processed sequences were aligned to the mouse genome assembly (GRCm38) using STAR and gene counts were extracted. Differential gene expression analysis was performed using a standard DESeq2 (v1.46.0) pipeline^80^ loaded in R^81^ (v4.5.0). Genes with log2(fold change) ≥ 0.58, and adjusted P-value ≤ 0.05 were considered significant. Gene Ontology(GO) terms of biological processes and pathway analysis were obtained using the DAVID functional annotation tool^82,83^, and dotplots for top enriched (P-value ranked) GO terms were constructed using clusterProfiler package (v4.14.4). Unsupervised k-means clustering and heatmap visualization was carried out using the ComplexHeatmap package^84^ using z-score normalized gene counts. For comparison with T2D datasets, human orthologues of mouse RNASeq data were generated using the Ensembl biomaRt webtool^85^. List of differentially expressed genes for *Gpt2*^βKO^ vs *Gpt2*^f/f^ are provided in Supplementary Tables 1 (control, 11mM glucose) and 2 (GLT).

### Western Blot Analysis

HEK293T cells and islets were collected and washed twice in PBS at 4°C, pelleted at 300–800g and resuspended in RIPA buffer (150 mM NaCl, 50 mM Tris-HCl, 1% NP-40, 0.5% sodium deoxycholate, 0.1% SDS) with added protease and phosphatase inhibitors (Millipore Sigma, 539134 and 524625–1SET), and sonicated at highest power for 4–6 30s sonication: cooling cycles (Bioruptor Plus, Diagenode). Supernatants were collected after 20 minutes of centrifugation at 13000g, 4°C, and protein was quantified (BCA assay kit; Thermo Fisher, PI23235). Six-30 μg of protein lysates was mixed with sample loading buffer (Invitrogen, NP0007) and proteins were separated by 10 or 12% denaturing SDS-PAGE gel electrophoresis, followed by transfer to nitrocellulose membranes and blocking in 1X PBST + 5% blotting-grade blocker (Biorad, 1706404). Membranes were incubated with primary antisera overnight at 4°C, and HRP conjugated secondary antibody the next day for 2 hours at room temperature. After incubation with HRP substrate (Millipore, WBLUR0100), blots were visualized on a Chemi-doc Touch Imaging system (Bio-Rad) and densitometric quantification was performed using Fiji^86^. Details for primary and secondary antisera are provided in Supplementary Table 3.

### Statistics

Values are reported as mean ±SE. To determine statistical significance between two groups, unpaired student’s t-test was used. One-way or two-way ANOVA with Tukey’s post hoc correction was used when comparing among multiple groups. P-value ≤ 0.05 was considered the threshold for considering significance. For performing hypergeometric test, the phyper function in R studio (v2024.09.0) was used and datasets showing FDR ≤ 0.05 were considered to have significant overlap. R studio (v2024.09.0) was used to create dot-plots and heatmaps. For all other statistics and graph plotting, GraphPad Prism v10 was used.

## Supplementary Files

This is a list of supplementary files associated with this preprint. Click to download.

- SupplementarytablesGpt2MSFinal2.xlsx
- Supplementary guresGpt2MSFinal2.docx
- 300dpiimages.pdf

## Data Availability

Raw data from the RNA sequencing experiment are being deposited in Gene Expression Omnibus, and accession number will be made available before publication. List of differentially expressed genes used in the study are provided as supplementary table and as CSV files in Figshare, along with high resolution images of each figure used in the main manuscript.
